# Dr. Walter Vaughan on Head-Aches and Their Cure

**Published:** 1832-01-01

**Authors:** 


An Essay on Headachs, and on their Cure. By *Walter Vaughan*, M.D. of the Royal College of Physicians in London. Octavo, pp. 252. London, Longman and Co.*

Here we have a work professedly written on a single symptom;—not indeed a symptom of one but of many diseases; and, if such a practice were to become general, medical books would increase, not in arithmetical but geometrical progression. Undoubtedly every author has a right to chuse his subject, and his manner of illustrating it; yet still he exercises the right at his peril, since eccentricity of design, and singularity of execution always challenge comment, and present little to avert reproof. But from us Dr. Vaughan has nothing to fear; his age entitles him to respect; his anxiety for the improvement of medicine is highly creditable to him; and his minute acquaintance with ancient and modern literature must for ever rank him among respectable and well-educated physicians. We will do him ample justice, and when we cannot praise we will not pain.

To write a book on a symptom, or even symptoms, of one or more diseases, is certainly a novel mode of proceeding, and for which there are but few precedents. It has been well remarked by a recent writer, that the symptoms of a disease do not constitute the disease itself;—they are rather

* The foregoing review was received a long time ago from one of our most esteemed and talented correspondents. We learnt at the time that the author of the work reviewed was labouring at once under mental anguish, bodily affliction, and reverses of fortune! Such being the case, we suspended the publication of the above critique, lest we should add to the wretched author's sufferings. But he is now gone to that land where criticism can never be felt—where flattery cannot soothe, nor censure wound the ear! Judgment may therefore be fairly pronounced on the book, without any injury to its far distant author.—Ed.

an algebraical character designating an unknown quantity, but which, in the hands of a skilful mathematician, may be managed as readily in working a proposition as if such unknown quantity were a sensible object. Let us take the disease, designated pneumonia, for the sake of farther illustration. The leading symptoms are thoracic pain and difficult breathing, while the disease itself is inflammation of the lungs. What would we think of an author, who, laudably anxious to elucidate this, or any other disease exhibiting such indications, should voluntarily write upon these indications in preference to the causes which gave them origin? To such a man we would say, you have forsaken the substance for the shadow; clung to effects and disregarded causes; and, as far as in your power laid, have inverted the natural order of sound observation and philosophical induction:—you resemble a hot-headed enthusiast, who contents himself with recording the phenomena of a diseased organ, when it is in his ability, and demanded by his duty to examine that organ with his own hands, and by his own eyes! Yet this, *mutatis mutandis*, Dr. Vaughan has designedly done, and, as we conceive, for no other apparent purpose than to gratify an eccentric spirit, and to display a comparatively extensive reading. Headachs, headachs are the eternal burthen of his singular, varied, and learned song, while, with all the enthusiasm of a heated imagination, and all the confidence, which a long life ought to create, he partly overlooks, or nearly disregards those great and interesting diseases—diseases which, to explain require the highest intellectual powers. How different is the design of Dr. Vaughan from the recommendation of a recent writer on practical medicine, Dr. Dawson, whose opinions so completely accord with our own, as to induce us to quote his language:—"Whenever individuals suffer from pain in the head, the attention should be directed to the discovery of its cause. It may be impending apoplexy or palsy; a spiculum of bone irritating or pressing upon the dura mater and other membranes, as well as the brain itself;—or it may be a harmless symptom of a disordered stomach."*

We will now attempt briefly to explain the nature of this Essay on Headachs: for it is works of a truly practical kind only that we distinguish by analysis; and, whatever may be the merits of this Essay in other respects, it certainly has little pretensions to that species of knowledge. Give us sterling facts, and we receive them thankfully; reason from these philosophically, and we listen with all the ardour of youth; raise an able superstructure on them, and we will cheerfully lend our humble aid; but it would be trifling with that public, which treats our labours so kindly, were we to trespass on its indulgence by loading our pages with long, tedious, and numerous extracts from almost innumerable authors of every age and country. Such is, in truth, the principal part of this Essay, interspersed with casual, and too often inappropriate remarks, which are indicative rather of singularity of opinion than of long experience. As Marc Anthony lost the world for a woman, and as Shakspeare frequently spoiled his finest passages for a paltry quibble, so does this writer sacrifice enlarged views, consistency of argument, and lucid connexion, for the sake of a trite observation, or a stale remark from authors consecrated to fame, because they fortunately

* Dawson's Practice of Physic, p. 289.

lived in an ancient and classic age. There is a time, especially when advanced years have triumphed over the romantic prepossessions of ardent youth, at which men ought to rise above the petty allurements of a name; and having outlived the gew-gaws of nominal authority they should throw away the rattle, and think for themselves. Far be it from us to depreciate the illustrious dead, we reverence their memory and respect their opinions, but we conceive their works to be the common property of all; and that he who writes a book should principally draw from his own stores, and not encumber his every page with long, and well known, extracts, like the idle pageants of a triumphal procession.

The Essay is dedicated to the Earl of Darnley, and the dedication informs the world of, what we believe no one questioned, the piety which exists at Cobham Hall. From the preface we learn the writer was perpetual pupil to Dr. Saunders (Lecturer on Medicine at Guy's Hospital) in the year 1784; and, in that preface, he thus explains his objects.

"1. To remove all ambiguity from the term Headach, by pointing out what is essential to the disease signified by that term, and what is not essential:

2. To show that there is a distinction of Headachs in the nature of things; and accordingly to make a division of them so perfect as to comprehend them all; that such errors of judgment as have too often arisen from the confounding of mere pains in the Head with Headachs, and different Headachs one with another, may in future be avoided: and

3. To give an enumeration of the most common occasions, on which Headachs take place; so as to trace out those principles, resting not on hypotheses, but on facts, upon which, as *data*, all reasoning concerning the nature and cure of any Headach should proceed." x.

This book contains five chapters:—I. Review of Sauvages on Headachs.—II. Definition of Headach.—III. Symptoms of Headach.—IV. Kinds of Headach, with the predisponent and occasional causes.—V. Cure of Headachs. And now the curtain is drawn up, the prologue has been spoken, the audience is impatient, and we retire from the stage at the commencement of the first act of this critical drama, in which Dr. Vaughan plays the only character, and delivers, with becoming gravity and suitable dignity, the following soliloquy.

"As for the division of Headachs into bilious, nervous, spasmodic, gouty, rheumatic, &c. as every one of these epithets contains a hypothesis, which I do not understand, and which I am persuaded, nobody else does, I shall not enter into a formal refutation of it: but I would ask those, who affect to reason so consequentially of a bilious Headach, what they mean by it? Is it a Headach depending upon a redundance of bile, or upon an alteration of its qualities?* If it be, where in the body of a patient must the bile be, to produce a Headach? Bile, even cystic bile, in the stomach, produces not Headach, but vomiting: an

---

* "Ou donne le nom de maladies bilieuses aux affections qui dépendant de l'Abondance et quelquefois de l'Alteration des Qualites de la Bile. Nysten's Dictionnaire de Medecine, &c. I cannot help thinking that Dr. Nysten should have followed the French Academy; and that Dr. Johnson should not have brought forward Spencer to sanction a manifest Error. '*Abundare* pro *redundare, supervacaneum,* seu *superfluum esse* perperam ponitur: nam *abundare,* copiam significat, non superfluitatem: adfert satietatem; non nauseam.' Nolten, Lexicon Antibarb."

excess of bile in the intestines produces not Headach, but Diarrhœa: bile in the blood might be supposed to produce a Headach, if pain in the head were a symptom of jaundice. But I do not think it proved, that bile ever enters the blood, either by regurgitating in the hepatic veins, or by absorption from the excretory ducts of the liver; first, because bile is never found in the lacteals, and never imparts its colour, or its taste to the chyle: secondly, because a jaundice arises sometimes in an instant from anger, in the twinkling of an eye from the bite of a viper, says Cardan;* therefore, sooner than its regurgitation, or its absorption can take place: thirdly, because one-half of the body, and sometimes only one extremity is jaundiced, which it could scarcely be, if bile were generally diffused in the blood: fourthly, because there is a yellow colour in the white of the eye, in the skin, in the expectoration at the end of peripneumony, and sometimes in scurvy, although there is no disorder of the liver: fifthly, because the skin is yellow after Ecchymoses: sixthly, because the matter discharged by vomiting and purging in cholera, and in yellow fever, is at length ascertained to be not bile, but some part of the blood exhaled and modified in a particular manner: and seventhly, because the serum of the blood, in the jaundice of new-born babes, contains none of the principles of bile. But they, who would know more of this subject, may consult M. M. Breschet, Desmoulins, and Lassaigne. To me it seems plain, that jaundice depends upon the blood itself.

And what is a nervous Headach? or rather, what Headach is not nervous? for every disease may, according to the two Professors of Medicine, Whytt and Cullen, be in some sense called nervous. And I would ask, what Headach differs so much from others that the epithet nervous belongs exclusively to it? I believe that in all Headachs, the nervous system is affected, and the pain is produced, before there is any turgescence of blood-vessels, or any phenomenon cognizable by the senses. It appears to me, that, if the situation of the blood is an object with nature in the commencement of most diseases, there is an antecedent state of the nerves of that situation, upon which the state of blood-vessels depends. Nay, I know no reason, that I should not conclude, that even an apoplexy sometimes kills a patient, before there is any sanguineous congestion in his head." 95.

Dr. Vaughan having finished his soliloquy, and retired altogether from the stage, we venture to re-appear, and to attempt to fill the remainder of this first act. We think the most cursory observer will be struck with the flippant and unphilosophical manner in which he commences. He acknowledges that certain writers have divided "headachs into the bilious, nervous, spasmodic, gouty, and rheumatic," and asserts that "every one of these *epithets* (for he is partial to the language of poetry) *contains* (an epithet may express something, but how it can contain any thing far exceeds our slender comprehension) a *(an)* hypothesis which he does not understand, and which, he is persuaded, nobody else does;" and, therefore, he "shall not enter into a formal refutation of it." Is this the language of courtesy, or of a sensible enquirer after truth? Does this author imagine that the opinions and experience of able and observing men are thus to be treated? If he do, we tell him he is mistaken; and, while he entertains such sentiments, he will receive only neglect; and, as he well knows, neglect is more painful than censure. When this gentleman affirms he does "not think it proved that bile ever enters the blood, either by regurgitating in the hepatic veins, or by absorption from the excretory ducts of the liver," we are startled, even in this age of medical scepticism, and content ourselves, as in the preceding instance, with making him no reply.

* Morgagni, de Sedibus et Causis Morborum, &c. Epist. lix. § 36.

In expressing the observations on the Essay of Dr. Vaughan, which a sense of public duty has impelled us to make, we are most anxious not to be misunderstood. We object to the plan of it, we object to the mode of execution, and we still more strongly object to its disregard of lucid arrangement, and its obvious want of practical utility; but we have not said, nor do we now intimate, that the performance does him discredit, or diminishes his character as a respectable physician. Indeed the tenor, as well as the spirit, of our observations must have conveyed a very different impression; yet, to avert the possibility of any misconception, we have made this explicit declaration. The Essay will, we believe, be little noticed by the youthful and active members of the profession, but it may be interesting to those advanced in life, and especially to literary men in general, too often severe sufferers from headachs, who are commonly wishful to know the opinions of the ancients and moderns on these afflicting affections. From the favoured pupil of Dr. Saunders,* the happy inmate of Dr. Babington,† and the fortunate friend of Sir Astley Cooper, for such are Dr. Vaughan's high pretensions, accompanied also by an experience of at least forty-two years, better things might have been expected; but it is a melancholy and influential truth, that early advantages do not always lead to great results; that reading is not learning, nor information talents; and even if they were, that learning without analysis is unprofitable, and talents without judgment unavailing. He that is blessed with genius or distinguished by abilities, and remarkable for sound observation and patient investigation, may produce an admirable work, in utter disregard of all learning; just like the bold and adventurous soldier who leads his army into the thickest of the fight, and bravely conquers there, in total defiance of all the received precepts of military tactics. Hannibal led armies on to victory, in complete ignorance of the instructions of that conceited pedant who presumed afterwards to lecture in his presence; while Hunter, another Hannibal in his line, gave to the world productions, that will only perish with the language in which they are written.

---

### VII.

### On Hyperæmia. By *M. Andral.*

M. Andral's work contains a mine of information, research, and deep thought, which the translation of Drs. Townsend and West have laid open to the mere English reader—provided they take the trouble to explore it.

---

* "Till his death, he continued to shew a warm and lively interest for my success in life, to honour me with his correspondence, and seldom to possess a book, domestic or foreign, which he did not lend me. He sent for me to Enfield after his retirement there. Need I say that I loved and reverenced him as a father?"—*Preface, page* 9.

† "With whom, in the early part of my life, I had the good fortune to be an inmate, and to whom I am indebted for much that I know."—*Page* 163.

